# Trends in Hip Fracture-Related Mortality Among Older Adults in the United States From 1999 to 2023: A Centers for Disease Control and Prevention’s Wide-Ranging Online Data for Epidemiologic Research (CDC WONDER) Analysis

**DOI:** 10.7759/cureus.90305

**Published:** 2025-08-17

**Authors:** Muhammad Tayyab, Muhammad Tanveer, Zawar Ahmad, Ameer Afzal Khan, Rahman Syed, Muhammad Shabir, Anfal Khan, Asif Afridi, Mohsin Ali, Fazal Syed

**Affiliations:** 1 Department of Trauma and Orthopaedics, Bradford Teaching Hospitals, Bradford, GBR; 2 Department of Trauma and Orthopaedics, Royal Stoke University Hospital, Stafford, GBR; 3 Department of Orthopaedics and Traumatology, Kettering General Hospital, Kettering, GBR; 4 Department of Internal Medicine, Saidu Medical College, Swat, PAK; 5 Department of Internal Medicine, Swat Medical College, Swat, PAK; 6 Department of Orthopaedics and Traumatology, Saidu Teaching Hospital, Swat, PAK; 7 Department of Orthopaedics and Traumatology, Queen Elizabeth Hospital, Birmingham, GBR; 8 Department of Internal Medicine, North West School of Medicine, Peshawar, PAK

**Keywords:** cdc wonder, disparities, hip fracture, mortality trends, united states

## Abstract

Background and aim

Hip fractures are a major cause of morbidity and mortality among the aging U.S. population. Despite advances in preventive strategies and fracture management, mortality following hip fractures remains a critical public health concern. This study aimed to analyze the national trends in hip fracture-related mortality among adults aged 65 years and older in the United States from 1999 to 2023 using the Centers for Disease Control and Prevention's Wide-ranging Online Data for Epidemiologic Research (CDC WONDER) data.

Methods

We conducted a retrospective, population-based analysis using the CDC WONDER multiple cause-of-death database. Age-adjusted mortality rates (AAMRs) per 100,000 population were calculated using the direct method standardized to the 2000 U.S. population. Joinpoint regression analysis was used to calculate annual percent changes (APCs) and identify significant shifts in mortality trends by sex, race/ethnicity, urbanization, U.S. census region, and state.

Results

Between 1999 and 2023, there were 334,905 hip fracture-related deaths among adults aged ≥65 years. The overall AAMR declined from 37.07 in 1999 to 23.91 in 2023. A significant decrease was observed from 2002 to 2018 (annual percent change (APC): -2.85%; 95% confidence interval (CI): -3.02 to -2.69; *p*<0.001), with a more recent decline from 2021 to 2023 (APC: -4.37%; 95% CI: -8.23 to -0.33; *p*=0.036). Both men and women showed significant long-term mortality reductions, with men experiencing a steeper decline. Racial disparities were evident, with the largest declines among the American Indian or Alaska Native individuals (APC: -4.01%; *p*<0.001). Non-metropolitan areas had higher mortality than metropolitan areas. Regionally, the Midwest had the highest AAMRs, while Montana, Colorado, and Minnesota recorded the highest state-level rates in 2023.

Conclusion

Hip fracture-related mortality among older adults in the United States has declined significantly over the past two decades, though recent trends suggest a plateau in some subgroups. Persistent disparities by race, sex, geography, and urbanization highlight the need for targeted public health interventions and equitable access to post-fracture care across the aging population.

## Introduction

Hip fractures remain a major public health concern among older adults in the United States, contributing substantially to morbidity, mortality, and healthcare costs. Despite advances in surgical management and supportive care, mortality rates following hip fracture continue to be unacceptably high. These injuries primarily affect adults aged 65 and older and often signify underlying frailty, osteoporosis, and a high risk of falls, which further compound adverse outcomes [1,2].

Globally, the absolute number of hip fractures continues to rise due to population aging, even as age-adjusted incidence rates have stabilized or declined in some countries [2]. In the United States, data indicate a decline in age-adjusted hip fracture rates since the 1990s, yet the associated mortality remains significant [3]. One-year mortality following hip fracture ranges between 17% and 25%, with affected individuals exhibiting a three- to four-fold higher risk of death compared to age-matched peers [4,5].

The Centers for Disease Control and Prevention’s Wide-ranging Online Data for Epidemiologic Research (CDC WONDER) is a valuable tool for monitoring national mortality trends. This platform provides access to multiple-cause-of-death data, enabling a detailed assessment of conditions such as hip fracture-related deaths across demographic and geographic subgroups [6]. Prior studies utilizing this database have identified over 200,000 hip fracture-associated deaths in adults aged 65 and older between 1999 and 2013 [7].

Although some reports suggest modest improvements in mortality, for example, a decline from 3.76% in 2000 to 2.92% in 2015, hip fracture remains a leading cause of injury-related death in older adults [8]. These improvements may reflect better surgical techniques, perioperative care, and early mobilization. However, disparities persist in mortality by sex, race, comorbid burden, and access to rehabilitation [9-11]. Men, though less frequently affected by hip fracture, demonstrate higher mortality than women [12]. Factors contributing to poor outcomes include delayed surgical intervention, postoperative complications, inadequate rehabilitation, and pre-existing conditions such as cardiovascular disease or cognitive impairment [13,14].

Tracking long-term trends in hip fracture-related mortality is essential to guide clinical practices and public health interventions. This study uses the CDC WONDER data from 1999 to 2023 to assess the national patterns in hip fracture mortality among older adults, with the goal of informing future policy, healthcare delivery, and preventive strategies.

## Materials and methods

Study setting and population

The CDC WONDER database was used to get death certificate data for this descriptive analysis. The data were then evaluated for hip fracture-related mortality in older adults from 1999 to 2023 using codes from the International Statistical Classification of Diseases and Related Health Problems, 10th Revision (ICD-10) as follows: S72.0 (femur neck fracture), S72.1 (peri-trochanteric fracture), and S72.2 (subtrochanteric fracture). Historically, hip fractures were identified in administrative databases using the same ICD codes [3,15]. It includes the cause-of-death information from death certificates for all 50 states and the District of Columbia [6]. The Multiple Cause-of-Death Public Use record death certificates were analyzed to identify hip fracture-related deaths. These were characterized as those with hip fractures stated as an underlying or contributing cause. People 65 years or older at the time of death were classified as older adults. Previous studies have defined older people using a similar age criterion [16,17]. Because this study used de-identified government-issued public use data and followed the STROBE (Strengthening the Reporting of Observational Studies in Epidemiology) reporting guidelines, it was exempt from local institutional review board approval. Data extraction and analysis were conducted in May and June 2025.

Data abstraction

Demographics, urban-rural classification, states, area, year, population size, and location of death were among the abstracted data. Demographics included gender, age, and race/ethnicity; places of death included home, hospice, nursing home/long-term care facility, and medical institutions (outpatient, emergency room, inpatient, death on arrival, or status unknown). Race and ethnicity were classified as Non-Hispanic (NH) White, NH Black or African American, Hispanic or Latino, NH American Indian or Alaska Native, and NH Asian or Pacific Islander. This information was previously used in WONDER database analysis and is based on death certificate data. The 2013 U.S. census classified counties as urban, large metropolitan area (population ≥1 million), medium/small metropolitan area (population 50,000-999,999), or rural (population <50,000) using the National Center for Health Statistics Urban-Rural Classification Scheme [18]. The Census Bureau classified the regions as Northeast, Midwest, South, and West [19].

Statistical analysis

To examine the national changes in hip fracture-related mortality, we calculated crude and age-adjusted mortality rates (AAMRs) per 10,000 population by year, sex, race/ethnicity, state, and urban-rural status, with 95% confidence intervals (CIs), from 1999 to 2023. The crude mortality rates were calculated by dividing the number of hip fracture deaths by the matched U.S. population for that year. To quantify national yearly trends in hip fracture-related mortality, the annual percent change (APC) with 95% CI in AAMR was determined using the Joinpoint trend analysis software (National Cancer Institute) [20,21]. This technique fits log-linear regression models when temporal variation occurs in order to detect significant changes in AAMR across time. Using two-tailed t-testing, APCs were classified as rising or declining if the slope of the change in mortality was significantly different from zero. P-values <0.05 were considered statistically significant.

## Results

**Figure 1:**
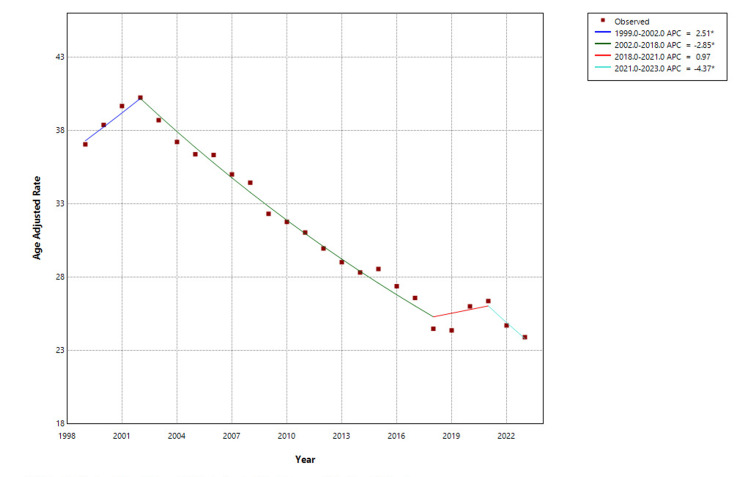
Overall hip fracture-related mortality in older adults, age-adjusted mortality rates (AAMRs) per 100,000 in the United States, 1999–2023. *indicates statistically significant APC (p<0.05). Abbreviations: APC=annual percent change. Created by Dr. Rahman Syed from the Centers for Disease Control and Prevention’s Wide-Ranging Online Data for Epidemiologic Research (CDC WONDER) data using Joinpoint trend analysis software.

From 1999 to 2023, a total of 334,905 hip fracture-related deaths were recorded among adults aged 65 years and older in the United States. AAMR declined markedly over the study period, decreasing from 37.07 per 100,000 in 1999 to 23.91 in 2023. Joinpoint regression analysis identified multiple trend phases. An initial modest increase in mortality occurred from 1999 to 2002, with an APC of 2.51% (95% CI: 0.44 to 4.62; p = 0.021). This was followed by a prolonged and significant decline from 2002 to 2018 (APC: -2.85%, 95% CI: -3.02 to -2.69; p<0.001). Between 2018 and 2021, the trend plateaued (APC: 0.97%, 95% CI: -3.07 to 5.18; p=0.620), but a subsequent decrease was observed from 2021 to 2023, with a statistically significant APC of -4.37% (95% CI: -8.23 to -0.33; p=0.036) as shown in Figure 1 (and Table 1 in Appendices).

**Figure 2:**
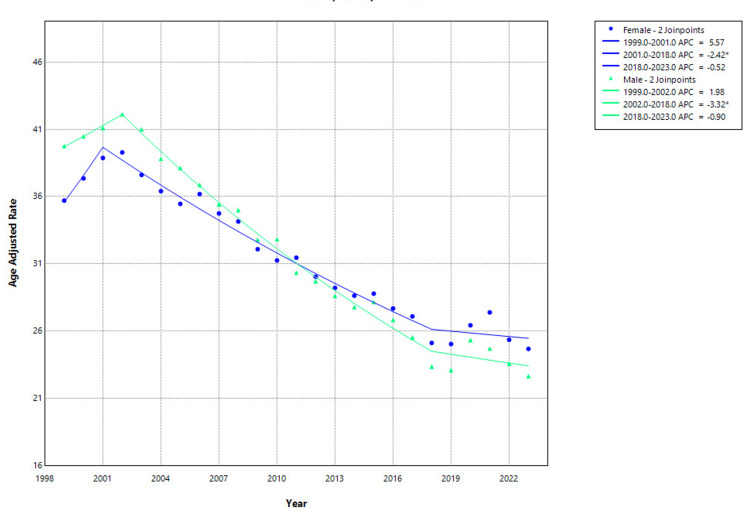
Gender-stratified hip fracture-related mortality in older adults, AAMRs per 100,000 in the United States, 1999–2023. *indicates statistically significant APC (p<0.05). AAMR=age-adjusted mortality rate; APC: annual percentage change. Created by Dr. Rahman Syed from the Centers for Disease Control and Prevention’s Wide-Ranging Online Data for Epidemiologic Research (CDC WONDER) data using Joinpoint trend analysis software.

Sex-stratified analysis revealed consistent declines in hip fracture-related mortality among both men and women, although temporal patterns varied. Among women, the AAMR decreased from 35.70 per 100,000 in 1999 to 24.69 in 2023. After a nonsignificant increase from 1999 to 2001 (APC: 5.57%, 95% CI: -1.92 to 13.63; p=0.138), mortality rates declined significantly from 2001 to 2018 (APC: -2.42%, 95% CI: -2.69 to -2.15; p<0.001). From 2018 to 2023, the trend remained stable (APC: -0.52%, 95% CI: -2.18 to 1.17; p=0.524). Among men, the AAMR decreased from 39.76 in 1999 to 22.66 in 2023. Although the initial trend from 1999 to 2002 was not statistically significant (APC: 1.98%, 95% CI: -2.01 to 6.12; p=0.315), a substantial and statistically significant decline was observed from 2002 to 2018 (APC: -3.32%, 95% CI: -3.64 to -3.01; p<0.001). From 2018 to 2023, the trend plateaued, showing a nonsignificant decline (APC: -0.90%, 95% CI: -2.62 to 0.84; p=0.287) as shown in Figure 2.

**Figure 3:**
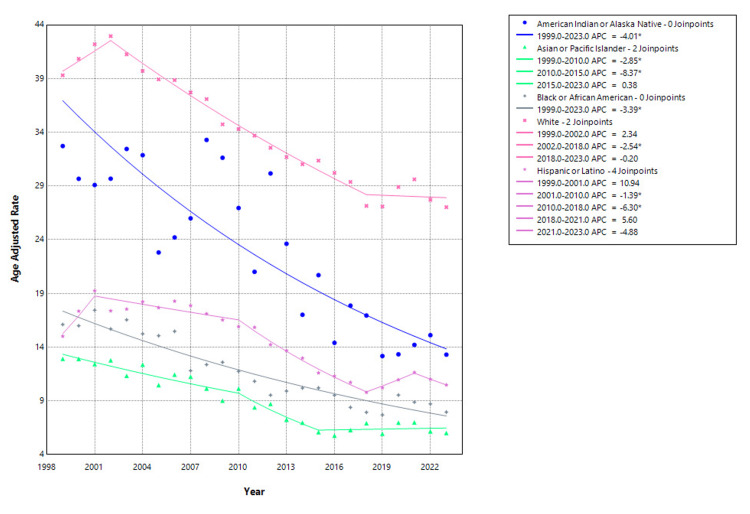
Race-stratified hip fracture-related mortality in older adults, AAMRs per 100,000 in the United States, 1999–2023. *indicates statistically significant APC (p<0.05). AAMR=age-adjusted mortality rate; APC=annual percentage change. Created by Dr. Rahman Syed from the Centers for Disease Control and Prevention’s Wide-Ranging Online Data for Epidemiologic Research (CDC WONDER) data using Joinpoint trend analysis software.

Racial and ethnic disparities were observed in hip fracture-related mortality trends. Among American Indian or Alaska Native individuals, the AAMR declined markedly from 32.71 per 100,000 in 1999 to 13.29 in 2023, with a statistically significant average APC of -4.01% (95% CI: -4.96 to -3.06; p<0.001). In the Asian or Pacific Islander population, AAMR decreased from 12.90 in 1999 to 6.00 in 2023, with significant declines from 1999 to 2010 (APC: -2.85%, 95% CI: -4.46 to -1.21; p=0.002) and 2010 to 2015 (APC: -8.37%, 95% CI: -14.47 to -1.84; p=0.016), followed by a nonsignificant trend from 2015 to 2023 (APC: 0.38%, 95% CI: -1.76 to 2.58; p=0.713). Among Black or African American adults, the AAMR fell from 16.10 in 1999 to 7.94 in 2023, with a significant decline over the entire period (APC: -3.39%, 95% CI: -3.85 to -2.92; p<0.001). In White adults, mortality declined from 39.30 to 27.02, with a non-significant increase between 1999 and 2002 (APC: 2.34%, 95% CI: -1.07 to 5.87; p=0.168), followed by a significant decline from 2002 to 2018 (APC: -2.54%, 95% CI: -2.82 to -2.26; p<0.001), and a plateau from 2018 to 2023 (APC: -0.20%, 95% CI: -1.76 to 1.38; p=0.788). In the Hispanic or Latino population, AAMR decreased from 15.00 in 1999 to 10.47 in 2023, though with variable trends. After a non-significant rise from 1999 to 2001 (APC: 10.94%, 95% CI: -1.05 to 24.38; p=0.071), the rate declined from 2001 to 2010 (APC: -1.39%, 95% CI: -2.39 to -0.39; p=0.011), followed by a steeper drop between 2010 and 2018 (APC: -6.30%, 95% CI: -7.32 to -5.26; p<0.001). More recent trends were mixed, with a non-significant rise from 2018 to 2021 and a subsequent decline from 2021 to 2023 that did not reach statistical significance, as shown in Figure 3.

**Figure 4:**
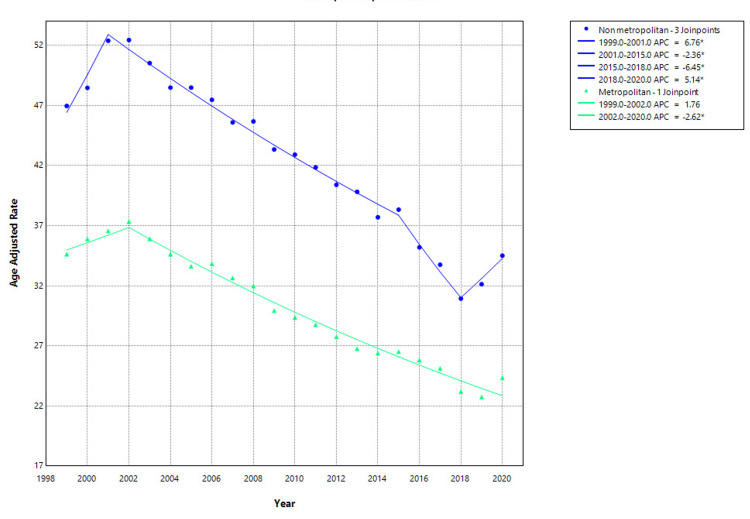
Urbanization-stratified hip fracture-related mortality in older adults, AAMRs per 100,000 in the United States, 1999–2020. *indicates statistically significant APC (p<0.05). AAMR=age-adjusted mortality rate; APC: annual percentage change. Created by Dr. Rahman Syed from the Centers for Disease Control and Prevention’s Wide-Ranging Online Data for Epidemiologic Research (CDC WONDER) data using Joinpoint trend analysis software.

Urbanization-specific analysis revealed significant differences in hip fracture-related mortality trends between metropolitan and non-metropolitan populations from 1999 to 2020. In non-metropolitan areas, the AAMR declined from 46.97 per 100,000 in 1999 to 34.51 in 2020, though with multiple trend shifts. A significant increase occurred from 1999 to 2001 (APC: 6.76%, 95% CI: 2.67 to 11.01; p=0.004), followed by a sustained decline from 2001 to 2015 (APC: -2.36%, 95% CI: -2.56 to -2.16; p<0.001). A steeper decline occurred between 2015 and 2018 (APC: -6.45%, 95% CI: -10.30 to -2.43; p=0.005), followed by a significant increase from 2018 to 2020 (APC: 5.14%, 95% CI: 0.86 to 9.60; p=0.022). In contrast, residents of metropolitan areas experienced a decline in AAMR from 34.63 in 1999 to 24.35 in 2020. After a non-significant change from 1999 to 2002 (APC: 1.76%, 95% CI: -1.98 to 5.64; p=0.339), a statistically significant and steady decrease followed from 2002 to 2020 (APC: -2.62%, 95% CI: -2.85 to -2.39; p<0.001) as shown in Figure 4. Urbanization-specific data beyond 2020 were not available.

**Figure 5:**
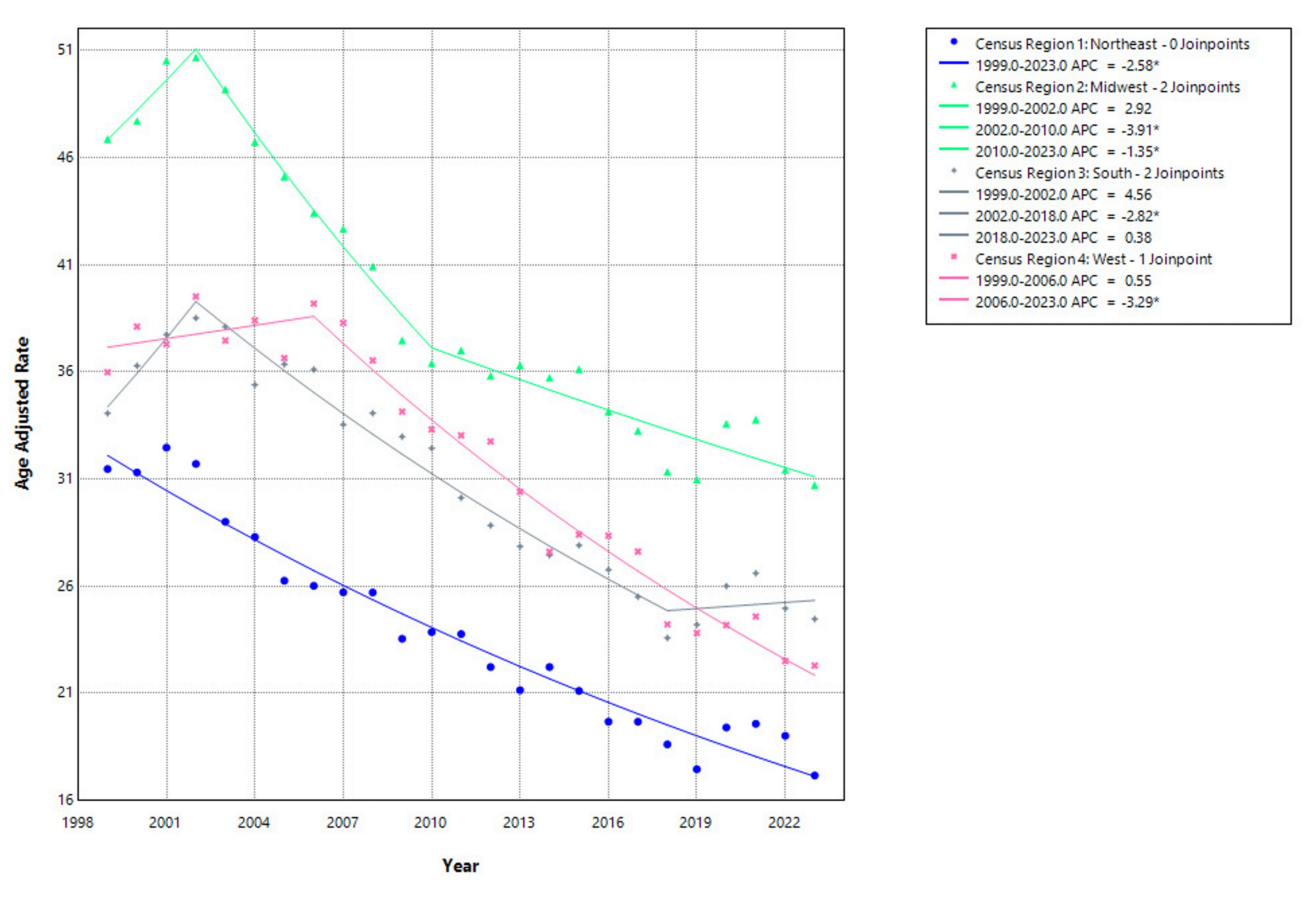
Census regions-stratified hip fracture-related mortality in older adults, AAMRs per 100,000 in the United States, 1999–2023. *indicates statistically significant APC (p<0.05). AAMR=age-adjusted mortality rate; APC=annual percentage change. Created by Dr. Rahman Syed from the Centers for Disease Control and Prevention’s Wide-Ranging Online Data for Epidemiologic Research (CDC WONDER) data using Joinpoint trend analysis software.

Regional analysis revealed consistent declines in hip fracture-related mortality across all four U.S. census regions from 1999 to 2023. In the Northeast, the AAMR declined from 31.47 per 100,000 in 1999 to 17.17 in 2023, with a statistically significant APC of -2.58% (95% CI: -2.83 to -2.34; p<0.001) over the entire study period. The Midwest experienced the highest overall burden, with AAMR falling from 46.87 to 30.72. After a non-significant rise from 1999 to 2002 (APC: 2.92%, 95% CI: -1.43 to 7.46; p=0.178), significant declines followed from 2002 to 2010 (APC: -3.91%, 95% CI: -5.02 to -2.78; p<0.001) and from 2010 to 2023 (APC: -1.35%, 95% CI: -1.83 to -0.87; p<0.001). In the South, AAMR decreased from 34.07 to 24.47, with a non-significant increase from 1999 to 2002 (APC: 4.56%, 95% CI: -0.25 to 9.60; p=0.062), a significant decline from 2002 to 2018 (APC: -2.82%, 95% CI: -3.19 to -2.45; p<0.001), and a plateau between 2018 and 2023 (APC: 0.38%, 95% CI: -1.62 to 2.43; p=0.695). In the West, mortality declined from 35.99 in 1999 to 22.30 in 2023. A non-significant trend was observed from 1999 to 2006 (APC: 0.55%, 95% CI: -0.86 to 1.97; p=0.429), followed by a significant drop from 2006 to 2023 (APC: -3.29%, 95% CI: -3.63 to -2.95; p<0.001) as shown in Figure 5.

**Figure 6:**
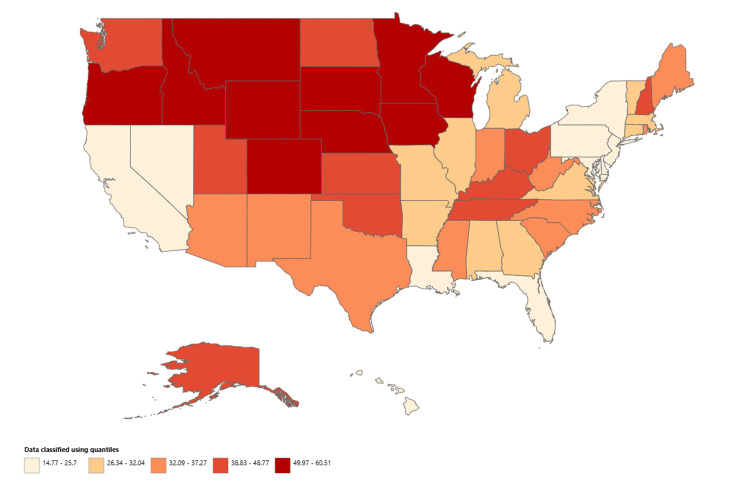
State-stratified hip fracture-related mortality in older adults, AAMRs per 100,000 in the United States, 1999-2023. AAMR: Age-adjusted mortality rate. Map created using the Centers for Disease Control and Prevention’s Wide-Ranging Online Data for Epidemiologic Research (CDC WONDER) data.

State-level analysis identified considerable geographic variation in hip fracture-related mortality among older adults in 2023. The seven states with the highest AAMRs were Montana (60.51 per 100,000), Colorado (60.20), Minnesota (59.80), Wisconsin (58.94), Wyoming (56.85), South Dakota (53.53), and Oregon (52.32), as shown in Figure 6.

## Discussion

**Figure 7:**
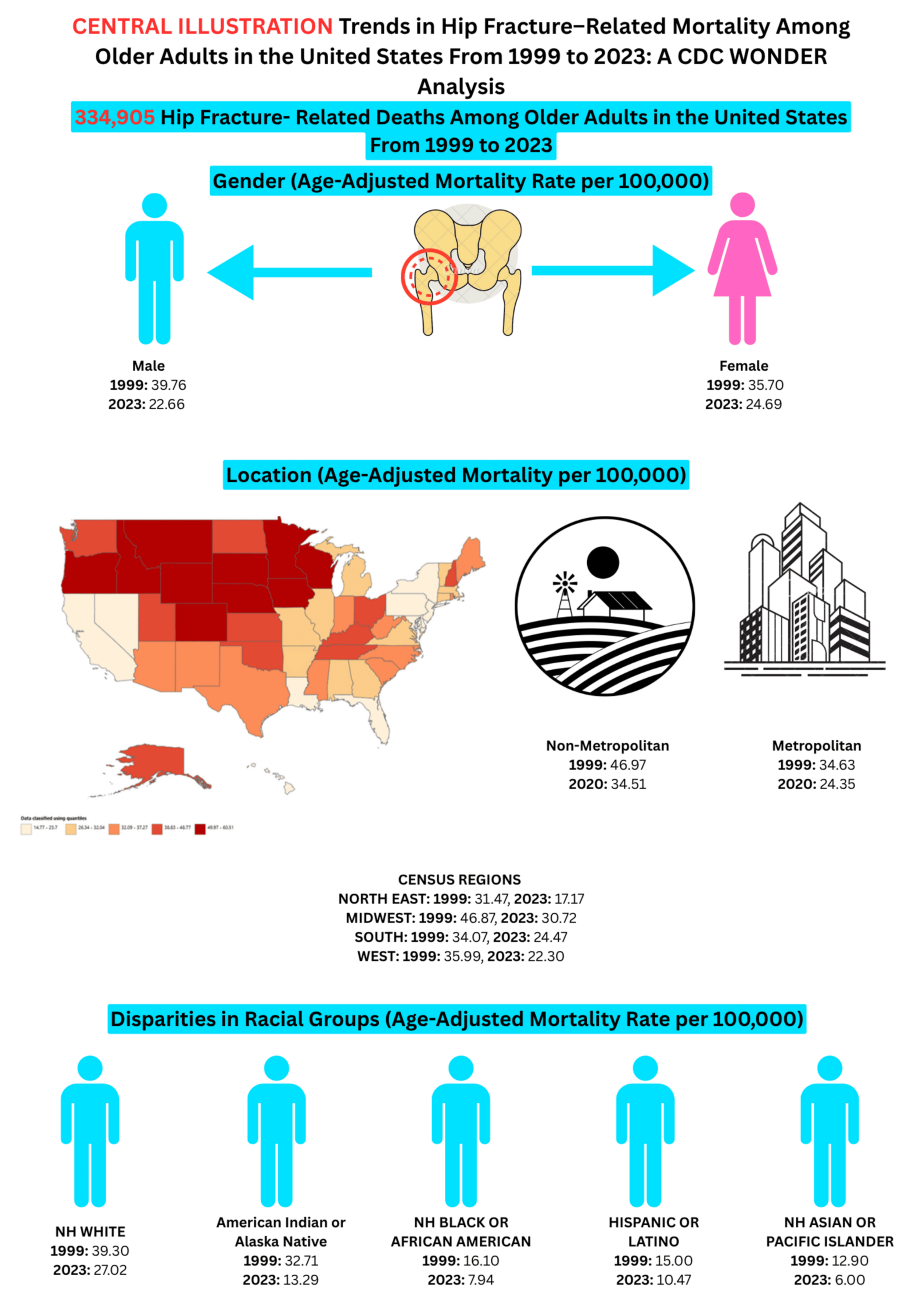
Central illustration Image credit: Created by Dr. Ameer Afzal Khan.

In this large-scale analysis of hip fracture-related mortality among U.S. adults aged 65 and older from 1999 to 2023, we observed a notable and steady decline in AAMRs from 37.07 per 100,000 in 1999 to 23.91 per 100,000 in 2023, as shown in a central illustration in Figure 7. That’s a 35.5% reduction over two decades, underscoring major strides in geriatric orthopedic care. These improvements echo international trends showing better outcomes for hip fractures over recent decades [2,22], which is particularly encouraging in light of the growing older population and rising global hip fracture burden [23].

Temporal trends and clinical implications

Our Joinpoint regression analysis highlighted distinct shifts in mortality trends: a mild uptick from 1999 to 2002, followed by a prolonged and significant decline through 2018, a plateau between 2018 and 2021, and then another decline after 2021. This timeline coincides with pivotal changes in clinical practice, such as early mobilization, streamlined surgical protocols, and team-based care models that gained traction in the early 2000s [24,25]. The drop in one-year mortality is in line with earlier reports, where rates fell from approximately 27% in the 1960s to 20% by the 2010s [26]. The durability of this improvement reflects deeper changes in care, faster time to surgery, modern anesthesia practices, and more robust perioperative strategies, which together have transformed the hip fracture care pathway [27,28].

Sex-based differences

Our findings also showed consistent differences by sex. While men had higher mortality rates throughout the study, they also experienced a steeper decline from 39.76 to 22.66 per 100,000 (a 43% drop), compared to a 31% decrease in women (35.70 to 24.69 per 100,000). These patterns reflect well-documented vulnerabilities in male patients after hip fractures, including more comorbidities and delayed treatment [29,30]. The greater improvement in male mortality could suggest that interventions have been especially beneficial for high-risk male patients. Still, men continue to have higher absolute mortality rates, pointing to the need for further targeted efforts to close this gap [31].

Racial and ethnic disparities

When looking at race and ethnicity, some striking patterns emerged. American Indian and Alaska Native populations saw the most dramatic improvement, with a 59% decline in mortality from 32.71 to 13.29 per 100,000. This may be due to community-level health initiatives or expanded access to orthopedic care [32]. In contrast, White adults had the highest overall mortality rates during the entire period, despite experiencing notable reductions. This finding deviates from usual health disparity trends, where minority groups often fare worse [33,34]. Meanwhile, Black, Hispanic, and Asian or Pacific Islander populations had consistently lower mortality rates. These differences could stem from varying fracture rates, bone health, healthcare usage, or reporting practices [35,36]. It’s important to interpret these trends carefully. Survival bias, healthcare access, and broader social determinants could be influencing both the incidence of fractures and the likelihood of mortality being recorded. More research is needed to untangle these complex dynamics [37].

Geographic and urban-rural variation

Our geographic analysis found that rural areas experienced consistently higher mortality rates than urban areas (46.97 vs. 34.63 per 100,000 in 1999). This gap is likely tied to known challenges in rural healthcare, including longer transport times, fewer specialists, and resource limitations [38]. Regionally, the Midwest bore the highest burden, while the Northeast achieved the lowest mortality rates by 2023. State-level differences were especially sharp, with Montana, Colorado, and Minnesota posting mortality rates above 59 per 100,000 in 2023. These regional patterns may reflect demographic trends, healthcare infrastructure, and even climate factors that influence fracture risk [39].

Limitations and future directions

This study has several limitations. First, reliance on death certificate data from the CDC WONDER database may result in underreporting or misclassification of hip fracture-related deaths, particularly in the presence of comorbidities. Second, the dataset lacks clinical details such as comorbid conditions, surgical treatment, time to intervention, and rehabilitation factors that significantly influence mortality outcomes. Third, race and ethnicity classifications may be inaccurate, and the absence of socioeconomic and behavioral data limits the analysis of health disparities. Urbanization data were unavailable beyond 2020, and small state populations may have introduced variability in mortality estimates.

Future research should incorporate linked clinical and administrative data to better understand the drivers of hip fracture-related mortality. Studies examining the impact of timely surgical care, post-fracture rehabilitation, and preventive strategies are needed. Additionally, regional and healthcare system-level evaluations could help identify best practices and care gaps. Expanding future analyses to include morbidity, functional outcomes, and the post-COVID-19 period will offer a more comprehensive understanding of trends in this high-risk population.

## Conclusions

Over the last 24 years, the United States has made real progress in reducing deaths from hip fractures among older adults. These improvements likely reflect a combination of clinical advances, better care coordination, and growing emphasis on evidence-based treatment. But we still have work to do. The remaining disparities based on sex, geography, and race or ethnicity are reminders that health equity remains an ongoing challenge. Continued focus on improving care delivery, especially in underserved populations, could drive even further gains in the years ahead.

## Appendices

**Table 1:** Overall hip-fracture deaths, AAMR, and Crude rate among older adults in the United States from 1999-2023 AAMR=Age-adjusted mortality rate

Overall	Year	Deaths	Crude Rate	Age-Adjusted Rate	Age-Adjusted Rate (Lower 95% Confidence Interval)	Age-Adjusted Rate (Upper 95% Confidence Interval)	Age-Adjusted Rate Standard Error
Overall	1999	12657	36.37	37.07	36.43	37.72	0.33
Overall	2000	13320	38.07	38.39	37.74	39.04	0.33
Overall	2001	13978	39.61	39.68	39.02	40.34	0.34
Overall	2002	14360	40.43	40.26	39.6	40.92	0.34
Overall	2003	14066	39.22	38.72	38.08	39.36	0.33
Overall	2004	13706	37.86	37.23	36.6	37.85	0.32
Overall	2005	13711	37.41	36.4	35.79	37.01	0.31
Overall	2006	14038	37.77	36.35	35.75	36.95	0.31
Overall	2007	13844	36.6	35.02	34.44	35.61	0.3
Overall	2008	13916	35.89	34.45	33.88	35.02	0.29
Overall	2009	13355	33.71	32.33	31.78	32.88	0.28
Overall	2010	13372	33.21	31.78	31.24	32.32	0.28
Overall	2011	13526	32.68	31.05	30.52	31.58	0.27
Overall	2012	13364	30.97	29.96	29.45	30.47	0.26
Overall	2013	13239	29.61	29.02	28.53	29.52	0.25
Overall	2014	13242	28.64	28.33	27.85	28.82	0.25
Overall	2015	13641	28.56	28.56	28.07	29.04	0.25
Overall	2016	13341	27.09	27.39	26.92	27.86	0.24
Overall	2017	13196	25.95	26.59	26.13	27.05	0.23
Overall	2018	12452	23.75	24.49	24.06	24.93	0.22
Overall	2019	12560	23.23	24.37	23.94	24.8	0.22
Overall	2020	13664	24.55	26.01	25.57	26.45	0.22
Overall	2021	12908	23.11	26.37	25.91	26.83	0.23
Overall	2022	13012	22.51	24.71	24.28	25.14	0.22
Overall	2023	12437	20.99	23.91	23.49	24.33	0.21
